# Pol γ possesses separate metal binding sites for polymerase and strand displacement functions

**DOI:** 10.1093/nar/gkag720

**Published:** 2026-07-20

**Authors:** Noe Baruch-Torres, Joon Park, Josue Mora-Garduño, Arkanil Roy, Anupam Singh, G Andrés Cisneros, Luis G Brieba, Smita S Patel, Y Whitney Yin

**Affiliations:** Department of Biochemistry and Molecular Biology, University of Texas Medical Branch, Galveston, TX 77555, United States; Sealy Center for Structural Biology and Molecular Biophysics, University of Texas Medical Branch, Galveston, TX 77555, United States; Department of Biochemistry and Molecular Biology, University of Texas Medical Branch, Galveston, TX 77555, United States; Sealy Center for Structural Biology and Molecular Biophysics, University of Texas Medical Branch, Galveston, TX 77555, United States; Unidad de Genómica Avanzada, Centro de Investigación y de Estudios Avanzados del IPN (CINVESTAV-IPN), Apartado Postal 629, CP 36821, Irapuato, Guanajuato, México; Department of Chemistry and Biochemistry, University of Texas at Dallas, Richardson, TX 75080, United States; Department of Biochemistry and Molecular Biology, Robert Wood Johnson Medical School, Rutgers University, Piscataway, NJ 08854, United States; Department of Chemistry and Biochemistry, University of Texas at Dallas, Richardson, TX 75080, United States; Department of Physics, University of Texas at Dallas, Richardson, TX 75080, United States; Unidad de Genómica Avanzada, Centro de Investigación y de Estudios Avanzados del IPN (CINVESTAV-IPN), Apartado Postal 629, CP 36821, Irapuato, Guanajuato, México; Department of Biochemistry and Molecular Biology, Robert Wood Johnson Medical School, Rutgers University, Piscataway, NJ 08854, United States; Department of Biochemistry and Molecular Biology, University of Texas Medical Branch, Galveston, TX 77555, United States; Sealy Center for Structural Biology and Molecular Biophysics, University of Texas Medical Branch, Galveston, TX 77555, United States

## Abstract

Accurate replication of the mitochondrial genome (mtDNA) depends on DNA polymerase γ (Pol γ), yet its strand-displacement activity has been reported with varying outcomes across studies. Here we show that human Pol γ carries out robust, processive strand-displacement synthesis under physiological divalent metal-ion concentrations. We identify two functional classes of metal-binding sites: high-affinity sites that support DNA synthesis and unwinding, and low-affinity sites that selectively suppress unwinding without impairing polymerase activity. Pol γ efficiently displaces DNA/DNA duplex and RNA/DNA hybrids, supporting a role in RNA primer removal during mtDNA replication. Cryo-EM structures of Pol γ bound to fork-mimicking DNA reveal conformational states corresponding to progressive duplex unwinding and identify structural elements that facilitate strand displacement. These findings establish a metal-dependent mechanism for Pol γ activity and reconcile previous discrepancies in its reported unwinding capacity.

## Graphical Abstract

**Graphical Abstract:**
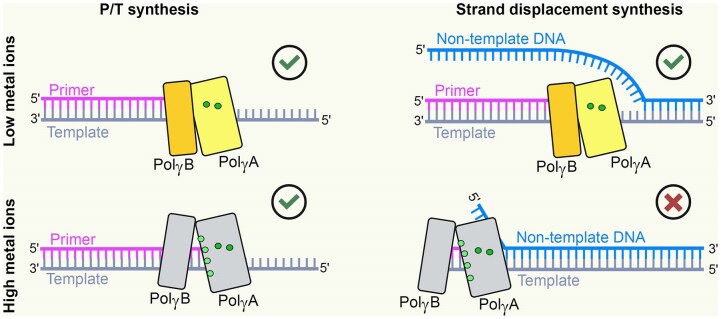

## Introduction

Mitochondrial DNA (mtDNA) encodes a subset of essential components of the oxidative phosphorylation electron transport chain, and its integrity is critical for cellular metabolism and energy production. Human mtDNA is a 16.6 kb double-stranded circular genome. The two strands (H- and L-strands) are replicated, depending on cell type, either by an asynchronous strand-displacement mechanism [1–3], in which the two strands are replicated asynchronously and proceeds asymmetrically, or by the RNA incorporated throughout the lagging strand strand-coupled (RITOLS) mechanism, which follows the canonical leading- and lagging-strand synthesis model [4–6].

Replication of double-stranded human mtDNA is carried out by a core replisome consisting of the Twinkle helicase, DNA polymerase γ (Pol γ), and mitochondrial single-stranded DNA-binding protein (mtSSB). In the classical division of labor within a replisome, the helicase unwinds duplex DNA using energy derived from ATP hydrolysis, while the DNA polymerase synthesizes DNA using the newly generated single-stranded DNA template. However, human Twinkle helicase alone exhibits weak DNA unwinding activity [7]. In contrast, Twinkle and Pol γ together can efficiently support duplex DNA unwinding and synthesis [8]. The observations raise the possibility that Pol γ may act as a driving molecular motor in the mitochondrial replisome by converting the chemical energy from dNTP incorporation into mechanical work to unwind the DNA duplex, an activity known as strand-displacement synthesis.

High-fidelity DNA polymerases that contain both polymerase (Pol) and exonuclease (Exo) active sites display a wide range of strand-displacement capabilities, from minimal to robust [9–14]. Because the termini of DNA duplexes are highly dynamic, resulting in 1–3 terminal base pairs fraying without external force [15], we define strand displacement synthesis here as the ability of a polymerase to extend beyond 5 bp into duplex DNA. A common observation is that exonuclease activity negatively impacts strand-displacement synthesis, as exonuclease-deficient DNA polymerases typically exhibit enhanced strand-displacement activity [9, 16, 17], suggesting the presence of an intrinsic motor function.

However, reports on the strand-displacement activity of Pol γ have been inconsistent, ranging from negligible to displacement of several dozen nucleotides [9, 18]. A single-molecule optical-tweezers study provided insight into this limited activity, showing that Pol γ generates sufficient forward force to unwind the first base pair of downstream duplex DNA but cannot prevent re-annealing of the displaced non-template strand. Consequently, unwinding of subsequent base pairs is reduced by ~30-fold [18]. Examination of prior studies suggests that the discrepancy in Pol γ strand displacement arises, at least in part, from differences in biochemical reaction conditions, specifically, the catalytic metal ion concentrations.

Here, we report systematic studies examining the dependence of Pol γ-mediated duplex DNA unwinding on divalent metal-ion concentrations. We show that, under physiological concentrations of Mg²⁺ or Mn²⁺, Pol γ displays robust strand-displacement synthesis with high fidelity and processivity up to 3–4 kb, whereas under nonphysiological conditions the activity is nearly completely suppressed. The underlying reason is that Pol γ contains at least two distinct putative metal-binding sites: high-affinity sites that support polymerase activity and DNA unwinding and low-affinity sites that suppress DNA unwinding without affecting DNA synthesis. Pol γ can displace RNA with comparable efficiency to DNA substrates. We determined cryo-EM structures of Pol γ bound to gapped duplex DNA that mimics a replication fork reveal several protein elements likely involved in strand-displacement activity. These findings highlight the importance of considering physiological reaction conditions when designing *in vitro* biochemical experiments. In the case of Pol γ, such conditions determine whether strand-displacement synthesis appears robust or merely marginal.

## Materials and methods

### Expression and purification of recombinant proteins

His-tagged Pol γA and Pol γB were expressed in *Sf9* insect cells and *E. coli* Rosetta (DE3) cells, respectively, and purified as previously described [19]. Briefly, both Pol γA wild-type and exonuclease-deficient variant (PolγA^exo−^) were purified to homogeneity using TALON (Cytiva, Marlborough, MA) and gel filtration column Superdex 200 columns. Pol γB was purified via Ni-NTA (Qiagen, Germantown, MD) and Mono S chromatography. Purified Pol γA and Pol γB were mixed at 1:2 ratio and purified on a Superdex 200 column. Fractions containing Pol γ holoenzyme were pooled, concentrated, and aliquoted for storage at −80°C.

T7 DNAP was purchased from New England Biolabs (catalog number M0274S).

### Nucleic acid substrates

The oligonucleotides were purchased from Integrated DNA Technologies, Inc. (Coralville, Iowa). A 5′-FAM-labeled 30-nt primer (ATT GGA AGT AGG GAT AGT CCC GAA CCT CGC) was annealed to a 74-nt template (5′-Biotin AGT GCT TAC ACC TGC CGC ATG ATC ACG GTA CGA GCT TGC TTT AGG CGA GGT TCG GGA CTA TCC CTA CTT CCA A/Inverted dT-3′) with or without the 85-nt non-template (5′-TTT TTT TTT TTT TTT TTT TTT TTT TTT TTT TTT TTT TTT TTT TTT AGC AAG CTC GTA CCG TGA TCA TGC GGC AGG TGT AAG CAC/Inverted dT-3′) to generate a p/t or a fork DNA.

The gap constructs containing DNA blocker or RNA blocker were formed by annealing a 40-nt DNA blocker (5′-AGC AAG CTC GTA CCG TGA TCA TGC GGC AGG TGT AAG CAC T-3′) or a 40-nt RNA blocker (5′-AGC AAG CUC GUA CCG UGA UCA UGC GGC AGG UGU AAG CACU-3′) to the 5′-FAM-labeled 30-nt primer/74 template.

Proofreading assays substrates were formed by annealing 5′-^32^P-labeled 26-nt primer, p26, (5′- AT ATT ATT TAC ATT GGC AGA TTC AAT-3′) to MM-Temp40 (5′-AA TCT AGT CCC AAG CTT GAA TCT GCC AAT GTA AAT AAT AT-3′) to a T·C mismatch containing p26/MM-Temp40 duplex at the 3′-OH end of the primer. MM-Temp40 was annealed to 5′-^32^P-MM-p40 (5′-^32^P-AT ATT ATT TAC ATT GGC AGA TTC AA**T** CTT GGG ACT AGA TT-3′) to form duplex MM-p40/MM-Temp40 with a mismatch, or to the 5′-^32^P-CM-p40 (5′-AT ATT ATT TAC ATT GGC AGA TTC AA**G** CTT GGG ACT AGA TT-3′) to form duplex CM-p40/MM-Temp40 without mismatch.

Circular 3.2 kb ssDNA was produced as reported previously with minor changes [20]. Briefly, 5 ml of 2× YT media supplemented with tetracycline/ampicillin was inoculated with XL1-Blue strain carrying pGEM3Zf(+)-HOM vector and incubated at 37°C for 12 h to increase the cell culture density. The culture was diluted with fresh 2× YT media containing VCSM13 helper bacteriophage (Agilent, Santa Clara, CA) at 6 × 10^7^ pfu/ml and incubated at 37°C for 1 h. To select the infected cells, kanamycin was added, and the incubation continued for another 18 h. The phage particles were precipitated and utilized for ssDNA extraction using E.Z.N.A.^®^ M13 DNA Mini Kit (Omega BIO-TEK), following manufacturer instructions.

The *midi*-*circle* DNA substrate was formed by annealing the 5′-Cy5 71-nt primer to the 3.2 kb circular ssDNA at 75°C for 5 min and then decreasing to 22°C at a rate of 1.0°C per 90 s in a Bio-Rad T100 thermocycler (Hercules, CA).

### Strand displacement assay on short fork DNA substrates

Two hundred nanomolar enzyme (holoenzyme Pol γ, Pol γ^exo–^, catalytic subunit Pol γA, or Pol γA^exo–^) was mixed with 100 nM DNA substrate in GB buffer (20 mM Hepes pH 8.0, 105 mM NaCl, 35 mM KCl, 2.5 mM DTT, 4% glycerol, 0.1 mg ml^−1^ BSA), DNA synthesis was initiated by addition of 0.2 mM dNTPs and Mg^2+^ or Mn^2+^ at indicated concentrations. The reaction mixture was incubated at 37°C for 10 min, then quenched by addition of Q buffer (80% formamide, 50 mM EDTA pH 8.0, 0.1% SDS, 5% glycerol, and 0.02% bromophenol blue). T7 DNAP was assayed under the same conditions except for the reaction buffer (20 mM Tris-Cl pH 7.5, 50 mM KCl, 1 mM DTT). The samples were quenched by heating at 95°C for 5 min, then cooled on ice, and applied to a 17% denaturing polyacrylamide gel with 8 M urea; the reaction products were separated by electrophoresis. The gel was imaged on a GE Typhoon 950 scanner and quantified using ImageQuant software TL (GE Healthcare). The percentage of full-length products was calculated as the intensity ratio of the full-length product to the sum of the total.

### Exonuclease assays

Reactions were initiated by the addition of 0.02 to 10 mM of Mg^2+^ or Mn^2+^ to mixture of 100 nM DNA substrate (p/t or fork DNA) and 200 nM Pol γ in GB buffer and quenched by adding 1:10 volume Q buffer after 10 min at 37°C. Quenched samples were boiled at 95°C for 5 min, cooled on ice, and loaded on a TBE 17% polyacrylamide gel containing 8 M urea. The gel was scanned using a GE Typhoon 950 imaging system. The band intensity corresponding to substrate and products for each Mg^2+^ concentration was quantified using the ImageQuant software TL (GE Healthcare). The percentage of primer degradation was calculated as the ratio of the remaining primer to the sum of the total band intensities.

### Proofreading assays

One hundred nanomolar of Pol γ was preincubated with 500 nM DNA duplexes p26/MM-Temp40, MM-p40/MM-Temp40, or CM-p40/MM-Temp40 in GB buffer (20 mM Hepes pH 8.0, 105 mM NaCl, 35 mM KCl, 2.5 mM DTT, 4% glycerol, 0.1 mg/ml^−1^ BSA) at 37°C for 5 min. The reaction was started by addition of 0.2 mM dNTPs and 0.32, 0.64, 1.25, 2.5, or 5 mM MgCl_2_. The reaction mixture was incubated for 20 min and quenched by adding 1:10 volume of Q buffer. A duplicated set of reactions was stopped by boiling at 95°C for 5 min and gradually cooled down to 20°C, followed by the HindIII digestion. Samples were boiled again at 95°C for 5 min and resolved on a TBE 17% polyacrylamide denaturing gel. DNA bands were quantified using ImageQuant software TL (GE Healthcare) and plotted as the percentage of cleaved products at each Mg^2+^ concentration.

### Unwinding RNA/DNA hybrid

A gapped construct mimicking when replisome reaches an upstream RNA primer was composed by annealing the 74-nt template to the 5′-FAM-labeled 30-nt primer and a 40-nt RNA blocker oligo. Primer extension assays were carried out with 200 nM of Pol γ and 100 nM of RNA or DNA blocker substrate in a GB buffer. Reactions were initiated at 37°C by the addition of 0.2 mM dNTPs and Mg^2+^ or Mn^2+^ at indicated concentrations. Reactions were stopped after 10 min incubation by addition of 1:10 volume Q buffer (80% formamide, 50 mM EDTA pH 8.0, 0.1% SDS, 5% glycerol, and 0.02% bromophenol blue). Reaction products were resolved on a 17% polyacrylamide denaturing gel, visualized on a GE Typhoon 950 scanner, and quantified using ImageQuant software TL (GE Healthcare). The percentage of full-length products was calculated by the intensity ratio of the full-length product to the sum of total intensities in the same lane.

### EMSA assay

One hundred nanomolar fork DNA was mixed with 200 nM holoenzyme Pol γ ^exo–^ or Pol γA ^exo–^ in a buffer containing 20 mM Hepes pH 8.0, 105 mM NaCl, 35 mM KCl, 2.5 mM DTT, 5% glycerol, and 0.1 mg/ml^−1^ BSA, incubated on ice for 5 min on ice, and then added the indicated Mg^2+^ concentrations (0.02–50 mM), and incubated at 37°C for 10 min. Samples were loaded on a 6% native polyacrylamide gel in a 0.5× TBE for 45 min at 180 V at 4°C. Gels were visualized by fluorescence in a GE Typhoon FLA 9000 Gel Scanner (Cytiva, Marlborough, MA).

### *In silico* simulation of Polγ divalent metal binding sites

The Pol γ system was modeled using the 4ZTZ crystal structure [21], focusing on the catalytic subunit Pol γA. The missing regions were filled with Rosetta Fold, generating multiple structures. The best model was selected based on visual inspection and RMSD alignment with the crystal structure as previously described [22]. Parameters for the incoming nucleotide were taken from prior work [23]. Protonation states were generated with ProPKA [24], at pH 7, and structural validation and hydrogen addition were performed using MolProbity [25]. The system was neutralized and solvated in AMBER18 with ff14SB [26], OL15 [27], and TIP3P force fields. Based on system volume, 72 Mg²⁺ ions and 144 Cl⁻ ions were added to achieve 20 mM MgCl₂, whereas three Mg²⁺ ions and 2 Cl⁻ ions were in the 1 mM system. Simulations were carried out for each of five created 1 mM systems to reduce bias. MD simulations were run in AMBER18 using pmemd.cuda [28]. Minimization involved 10 000 cycles (steepest descent and conjugate gradient). Heating to 300 K was done with Langevin dynamics [29] using a 2 ps⁻¹ collision frequency and initial restraints of 100 kcal mol⁻¹Å⁻², then gradually reduced. The systems with 20 mM concentrations had a production run of 500 ns in triplicates (for a total of 1.5 μs), and the 1 mM concentrations had a production of 100 ns in quintuplets due to the random placement of the ions (for a total of 500 ns). All bonds involving hydrogen were treated with SHAKE [27]. Long-range Coulomb interactions were handled with smooth Particle Mesh Ewald method [30], and long-range van der Waals interactions are approximated using default isotropic correction in AMBER with a 10 Å cutoff. Parameters used for calculations and starting coordinates were present in Zenodo (https://zenodo.org/records/4469899). Analysis of the simulations was done using the CPPTRAJ suite of AMBER.

### Metal competition binding assays

200 nM Pol γ was mixed with 100 nM DNA and incubated for 5 min at 37°C, 0.2 mM dNTPs, and Ca^2+^ (0 to 10 mM) with or without constant concentration 0.32 mM Mn^2+^ (or 0.64 mM Mg^2+^), and incubated for 10 min. The reaction mixtures were quenched with 1:10 volume Q buffer. Reaction products were resolved on a TBE 17% denaturing polyacrylamide gel, visualized on a GE Typhoon 950 scanner, and quantified using the ImageQuant software TL (GE Healthcare).

### Midi-circle template replication assays

Seven nanomolar 3.2 kb *midi-circle* DNA annealed to a Cy5 primer was mixed with 35 nM Pol γ in GB buffer for 10 min on ice and 5 min at 37°C before adding 0.2 mM dNTPs and 0 to 10 mM MgCl_2_. The reaction mixture was incubated for another 15 min, quenched by addition of 6× QB solution (18% Ficoll 400, 6% SDS, and 120 mM EDTA pH 8.0), and subjected to electrophoresis on a 0.8% alkaline gel at 20 V for 17 h. The gels were neutralized by soaking in AB buffer (1 M Tris-Cl, pH 7.6, 1.5 M NaCl) for 45 min in rocking motion, incubated with SYBR gold dye (1× final concentration) for 60 min in 1× TAE buffer, and scanned in a GE Typhoon 950 imaging system using dual-wavelength mode at 635 nm to visualize the Cy5 fluorophore on the primer and at 488 nm to visualize SYBR Gold-stained DNA.

### Cryo-EM sample preparation

A 77-nt dumbbell DNA (5′P-GCT TTT CTG GTG AAA AGC TGG TCG GCA GCG CTT GAG CAG CGG CAG CTG GTG CTG CCG CTG CTC AAG CGC TGC CGA C/ddC/-3′) was synthesized with 5′ phosphate and 3′ dideoxycytosine and purified with high-performance liquid chromatography by Integrated DNA Technologies. It was annealed by heating to 95°C for 5 min and slowly cooling overnight.

Two micromolar of Pol γ holoenzyme formed by Pol γA^exo–^ and Pol γB ΔI4 [31] was incubated on ice with an equimolar 77-nt dumbbell DNA in buffer containing 25 mM HEPES, pH 7.5, 140 mM KCl, 10 mM CaCl_2_, 10 mM βME, 0.01% octyl-β-glucoside, and 1 mM dATP. The sample (4 µl) was applied to a plasma-cleaned QUANTIFOIL R 2/1 Cu 200 grid and rapidly frozen in liquid ethane using a Vitrobot Mark IV system at 22°C and 100% humidity (Thermo Fisher Scientific).

### Cryo-EM data acquisition and processing

The frozen grids were loaded into a Titan Krios G3i (Thermo Fisher Scientific) equipped with K3 direct electron detector with BioQuantum energy filter (15-eV energy slit) (Gatan) operated at 300 keV at Stanford SLAC Cryo-EM Center. Cryo-EM data were automatically acquired using EPU software in super-resolution counted mode at a nominal magnification of ×105 000 (corresponds to 0.43 Å/pix) with a nominal defocus range between −1.5 and −2.5 μm. Forty-frame movie stacks were collected over 2-s exposure with a total dose of 49.60 e^−^/Å. A total of 10 227 movie data were collected.

The movie frames were imported into cryoSPARC [32] for image processing (Supplementary Fig. S1). Movies were motion-corrected and 2× binned using Patch Motion Correction, resulting in 0.86 Å/pix. Contrast transfer function (CTF) of resulting micrographs was determined using patch CTF estimation. After discarding micrographs with CTF fit higher than 4 Å, the remaining 9974 micrographs were denoised using the Micrograph Denoiser. 9 765 281 particles were picked and extracted with 4× binning from the denoised micrographs using the template picker. The 2D templates were created using EMD-27154. After iterative 2D classifications, 2 308 853 pruned particles were subjected to an initial 3D volume (volume 1) reconstruction using 579 000 particles with *Ab Initio* job as well as four amorphous 3D volumes (volumes 2–5) created by prematurely terminating another *Ab Initio* job. Resulting five 3D volumes were subjected to heterogeneous refinement, and 1 525 828 particles belonging to volume 1, the good initial 3D volume, were extracted without binning. Homogeneous refinement was applied to the selected particles to 2.80 Å. Another round of heterogeneous refinement was performed using the 3D volume from the previous homogeneous refinement as 3 repeated input volumes. 1 158 228 particles belonging to the resulting classes H0 and H1 were selected and refined to 2.91 Å. These particles were subjected to local and global CTF estimations [33] followed by a non-uniform refinement [22], resulting in a 2.73 Å second reconstruction. A reference-based motion correction was performed on the outputs of the previous non-uniform refinement, and the motion-corrected 1 157 747 particles were subjected to another non-uniform refinement, resulting in 2.59 Å reconstruction. 3D flexible refinement [34] was performed on 1 157 000 particles, which revealed two dominant molecular motions present in the dataset. To tease out different conformations relevant to the strand displacement activity of Pol γ, local refinement was first performed by creating a focused mask surrounding the catalytic subunit Pol γA and the downstream duplex DNA, and then the newly aligned particles were subjected to 3D classification with the same focused mask. Out of 10 classes, 4 classes were selected for further non-uniform refinement, resulting in 2.62 Å, 2.94 Å, 3.23 Å, and 3.05 Å reconstructions for classes C4, C2, C5, and C0, respectively, according to the gold standard FSC (GSFSC) at 0.143.

### Model building

Cryo-EM maps were post-processed with DeepEMhancer [35] and LocSpiral [36]. Pol γ ternary complex structure (PDB: 8D33) was used to build initial models. Manual adjustments were performed in Coot [37] and ISOLDE [38]. Atomic models were refined in real-space refinement in Phenix [39] using LocSpiral-processed maps. Refined models were validated with Molprobity and Q-score analysis [40]. Structural analysis was performed in PyMOL [41] and ChimeraX [42], and structural search was performed with Foldseek [43]. Figures were prepared using ChimeraX.

## Results

### Dependency of Pol γ strand displacement synthesis on divalent metal ion concentrations

**Figure 1.**
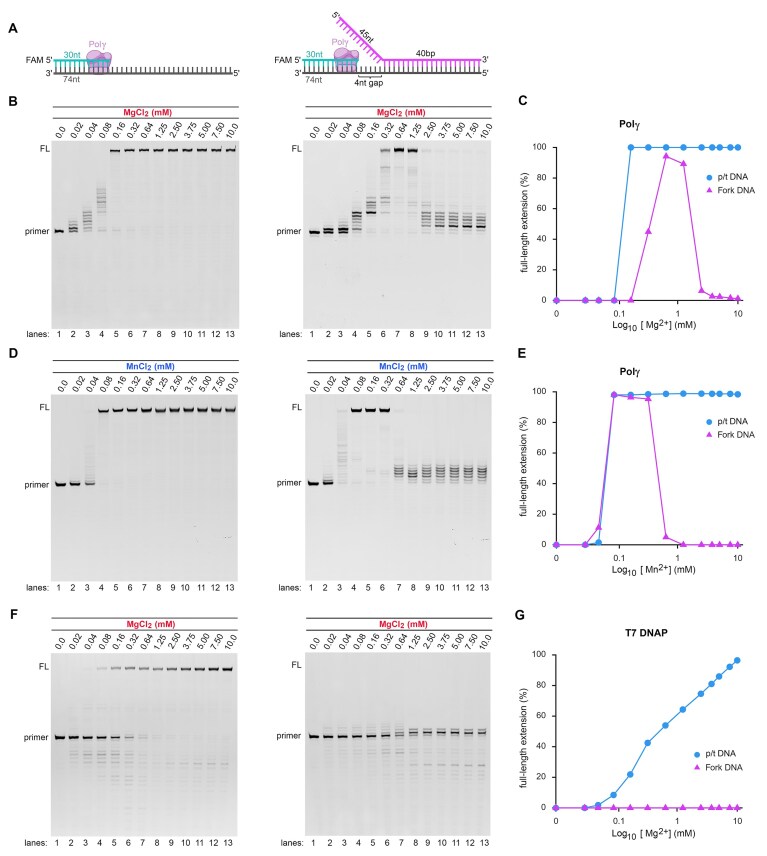
Pol γ metal-dependent DNA strand displacement synthesis activity. (**A**) Schemes of p/t (left) and short fork (right) DNA substrates, (**B**) Pol γ DNA synthesis on the p/t and fork DNA in the presence of 0 to 10 mM Mg^2+^, (**C**) quantification of percentage of full-length P + 44 versus Mg^2+^ concentration, (**D**) Pol γ strand displacement synthesis in the presence of 0 to 10 mM of Mn^2+^, and (**E**) quantification of panel (D). (**F**) similar experiments as in panel (B), except Pol γ is substituted by T7 DNAP, and (**G**) quantification of panel (F).

Biochemical studies on human Pol γ DNA synthesis activity have typically performed at catalytic metal ion, Mg^2+^ concentrations from 5 to 20 mM [8, 44–46], higher than the physiological level of 0.45–1.2 mM [47, 48]. Because results from these experiments showed varied Pol γ activities, we systematically measured Pol γ strand displacement synthesis across a range of 0.02–10 mM Mg^2+^ or Mn^2+^ concentrations on a fork DNA, which is formed by a 5′-FAM 30-nt primer and an 85-nt flap annealed to a 74-nt template, resulting a 40 bp downstream duplex, a 45-nt 5′-flap, and a 4-nt gap (Fig. 1A). Upon addition of dNTPs, Pol γ gap-filling synthesis and strand displacement synthesis can be simultaneously monitored by primer extension products to 4-nt (P + 4) and 44-nt (P + 44), respectively. The duplex of the same primer and the template (p/t) was formed for measuring DNA synthesis only (Fig. 1A).

On the p/t, Pol γ produced P + 44 beginning at 0.02 mM Mg^2+^ and continued up to 10 mM Mg^2+^ equivalently (Fig. 1B, *left panel*). In contrast, on the fork DNA, Pol γ strand displacement product P + 44 was observed only at a narrow [Mg^2+^] range: 0.32–1.25 mM (Fig. 1B, *right panel*). Meanwhile, the gap-filling synthesis remained invariable at higher metal concentrations (Fig. 1B, *right panel*). The results showed that Pol γ DNA synthesis is insensitive to Mg^2+^ concentration, while its DNA unwinding activity is highly sensitive to Mg^2+^ concentration (Fig. 1C).

Previous studies reporting different levels of Pol γ strand-displacement synthesis were conducted under varying Mg²⁺ concentrations. For example, minimal activity (1–2 nt) was observed at 10 mM Mg²⁺ [9], whereas moderate activity (22–52 nt) was reported at 4 mM Mg²⁺ [18]. Our results obtained under these conditions are consistent with these findings, suggesting that differences in Mg²⁺ concentration may contribute to the reported variability in Pol γ strand-displacement activity.

We next evaluated whether the metal dependency exists with another catalytic metal Mn^2+^. Again, Pol γ generated the strand displacement product P + 44 only with 0.08–0.32 mM Mn^2+^, which happens to be the physiological concentration range, and fell sharply at Mn^2+^ concentrations greater than 0.64 mM (Fig. 1D, *right panel*), while the synthesis on the p/t is invariable with Mn^2+^ concentration (Fig. 1D, *left panel* and 1E).

We examined the activities of the catalytic subunit Pol γA or PolγA^exo–^ and found that neither displayed strand displacement synthesis at any concentrations of Mg^2+^ or Mn^2+^ (Supplementary Fig. S2). Nonetheless, Pol γA generated gap-filling product at all concentrations of Mg^2+^, indicating Pol γA can bind to the fork DNA and simply lacks DNA unwinding capability. Although Pol γA displayed lower DNA-binding affinity [49], metal-dependent binding assay showed that it bound to the fork DNA under our experimental conditions (Supplementary Fig. S3B). The results indicate that formation of holoenzyme is essential for motor function.

To identify the source for the metal dependency, we examined whether the holoenzyme is disrupted at higher metal ion concentrations using EMSA. Pol γA and the holoenzyme binding to the same fork DNA were performed under identical conditions for activity assays. Pol γA alone was able to form 90% complex with the fork DNA; in the presence of the holoenzyme, a supershifted band was observed throughout the metal ion concentrations (Supplementary Fig. S3), indicating the holoenzyme is intact, and consistent with that Pol γ holoenzyme increases the catalytic subunit DNA binding affinity [49, 50].

We next examined whether the fork DNA’s downstream duplex is weakened at lower metal ion concentrations using wild-type T7 DNAP that is unable to continue synthesis on dsDNA [51]. If the downstream dsDNA of the fork is weakened, then T7 DNAP should produce the same metal-dependent strand displacement synthesis. While T7 DNAP synthesized the P + 44 product on the p/t at 0.08–10 mM Mg^2+^ (Fig. 1F, *left*), its strand displacement product P + 44 was completely lacking on the fork DNA (Fig. 1F, *right panel*), indicating the fork DNA’s downstream 40 bp remains duplex (Fig. 1G). Furthermore, the calculated melting temperature (T_m_) of the 40 bp DNA [52] is 74.4–75.4°C at 0.64–1.25 mM Mg^2+^, and 76.6–82.9°C at 2.5 mM-50 mM Mg^2+^ (Supplementary Table S1), thus greater than 99% of downstream DNA exists as a duplex under our experimental 37°C condition.

### Metal dependency of Pol γ exonuclease activity

Because silencing exonuclease activity greatly enhances Pol γ strand displacement activity [9], we thus evaluated whether the metal ion concentration that enables Pol γ strand displacement activity also suppresses the exonuclease activity.

We repeated the assays from 0 to 10 mM of Mg^2+^ or Mn^2+^. At 0.64–1.25 mM Mg^2+^, the concentration supporting strand displacement, Pol γ exonucleolytic products on the fork DNA or p/t DNA were 45%–70% of that at 10 mM Mg^2+^. In the presence of 0.16 and 0.32 mM Mn^2+^, Pol γ excised 90%–98% of the primer relative to that at 10 mM Mn^2+^ (Supplementary Fig. S4). The results clearly indicate that Pol γ exonuclease activity does not overlap with strand displacement in the presence of either Mg^2+^ or Mn^2+^ (Supplementary Fig. S4C and E), suggesting that the metal ion-regulated strand displacement is not entirely caused by reducing exonuclease activity.

To test the conclusion, we assayed Pol γ^exo–^ strand displacement synthesis metal dependency. The P + 44 products reached maximum quantity at 0.64–5.0 mM Mg^2+^ but declined at 7.5 mM and were diminished at 30 mM Mg^2+^, in contrast to the Pol activity on a p/t substrate that remained unchanged at 0.32–50 mM Mg^2+^ (Supplementary Fig. S5). Strikingly, in the presence of Mn^2+^, Pol γ^exo–^ strand displacement synthesis showed metal sensitivity comparable to that of Pol γ (Supplementary Fig. S5D–G), confirming metal regulation of Pol γ strand displacement via factors beyond exonuclease activity.

### Fidelity of Pol γ strand displacement

**Figure 2.**
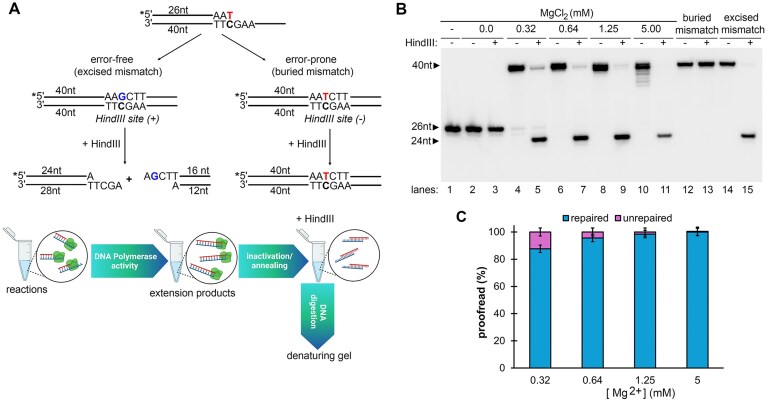
Pol γ mismatch proofreading under different Mg^2+^ concentrations. (**A**) Scheme of experimental design, (**B**) gel electrophoresis resolved primer extension products with and without Hind III digestion at the indicated Mg^2+^ concentrations. The 26-nt band is the primer, the 24-nt band is a product of Hind III digestion, and (**C**) quantification of percentage of proofread misincorporation at four Mg^2+^ concentrations. Blue bars indicate the percentage of proofread (24-nt) product, whereas pink bars indicate the percentage of non-proofread (40-nt) product. Percentages were calculated relative to the total signal in each lane. Error bars represent the standard deviation of three independent experiments.

The assay was conducted on a primer/template DNA with a T·C mismatch at the 3′-end of the primer, similar to a previous report [53]. If Pol γ proofreads the mismatch and resynthesizes with a correct nucleotide dGTP, a *HindIII* site will be formed; if Pol γ buries the mismatch without proofreading, no *Hind* III site will be formed (Fig. 2A). The ratio of *Hind* III cleaved versus noncleaved products was defined as proofreading efficiency. The controls, 40-nt DNA duplexes containing either a mismatch (T·C) or match (G·C) base pair, displayed 0% and 100% proofreading efficiency (Fig. 2B, *lanes* 12–15). In the presence of 0.32 mM, 0.64 mM, 1.25 mM, and 5.0 mM Mg^2+^, Pol γ proofreading efficiencies were 88%, 96%, 98%, and 100%, respectively (Fig. 2B, *lanes* 4–11 and 2C). The results suggest that the proofreading function is largely intact under conditions that Pol γ is capable of strand displacement synthesis.

### Strand displacement of RNA

**Figure 3.**
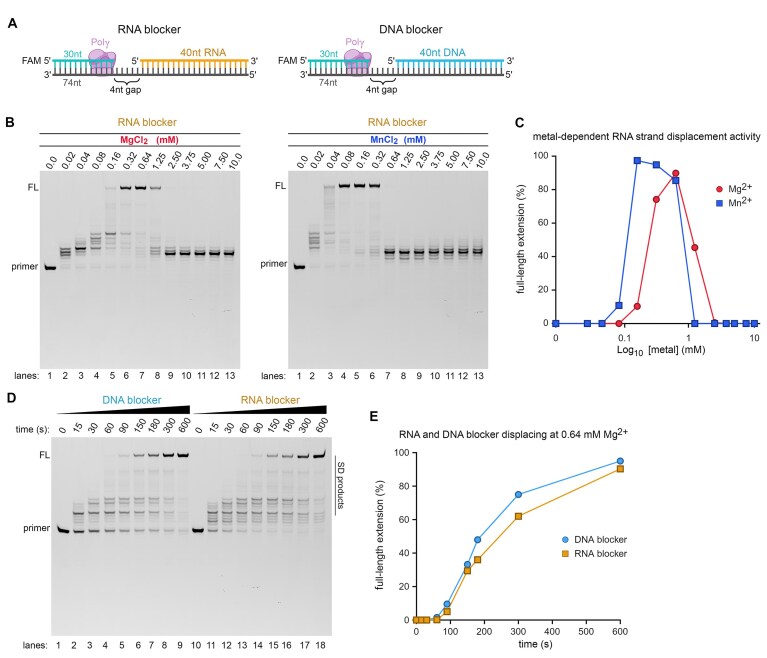
RNA and DNA primer removal by Pol γ. (**A**) Scheme of the RNA and DNA blocker substrates used in this assay containing a 4-nt gap and 40-bp downstream region. (**B**) RNA strand displacement assays were performed in a 0 to 10 mM gradient of Mg^2+^ or Mn^2+^ displaying a full-length product (FL) from 0.16 to 1.25 and 0.04 to 0.32 mM, respectively, and (**C**) quantification of extended products from panel (B). (**D, E**) time courses of DNA and RNA displacement syntheses and quantification.

The RNA primer that initiates replication needs to be removed and replaced with DNA. To test whether Pol γ can displace the RNA using its strand-displacement synthesis, we designed a gapped constructs containing a 74-nt template annealed to an upstream 5′-FAM-labeled 30-nt DNA and a downstream 40-nt RNA blocker or a DNA blocker (Fig. 3A). Pol γ completely displaced RNA and extended all primers to full-length at the same [Mg^2+^] as the fork DNA (Fig. 3B compared to Fig. 1B); and decreased the activity at [Mg^2+^]$\ge$2.5 mM. Similar behavior was observed in the presence of Mn^2+^, where robust RNA displacement synthesis occurred only at 0.08–0.32 mM Mn^2+^ (Fig. 3B and C). The RNA is displaced at a comparable rate as the DNA (Fig. 3D and E).

### Pol γ possesses distinct metal-binding sites for regulating DNA unwinding

The above results led us to hypothesize that Pol γ possesses at least two classes of metal binding sites: the high (H-) affinity sites that bind to Mg^2+^ at 160 µM–1.25 mM or Mn^2+^ at 80–320 µM, and low (L-) affinity sites that bind [Mg^2+^] ≥2.5 mM or [Mn^2+^] ≥0.64 mM. Metal ions bound in the H-sites support Pol γ catalyzing DNA polymerase and exonuclease activities, and promote dsDNA unwinding, whereas those bound to the L-sites inhibit DNA unwinding.

To confirm the existence of the two distinct affinity sites, we tested Pol γ H- and L-sites can be occupied by different metal ions and the effects on strand displacement activities. Specifically, we tested Pol γ activity in the presence of 0–10 mM Ca^2+^, a frequently used non-catalytic metal substitute that binds in the Pol and Exo active sites but impairs the activities in vast majority of DNA polymerases [54–58].

**Figure 4.**
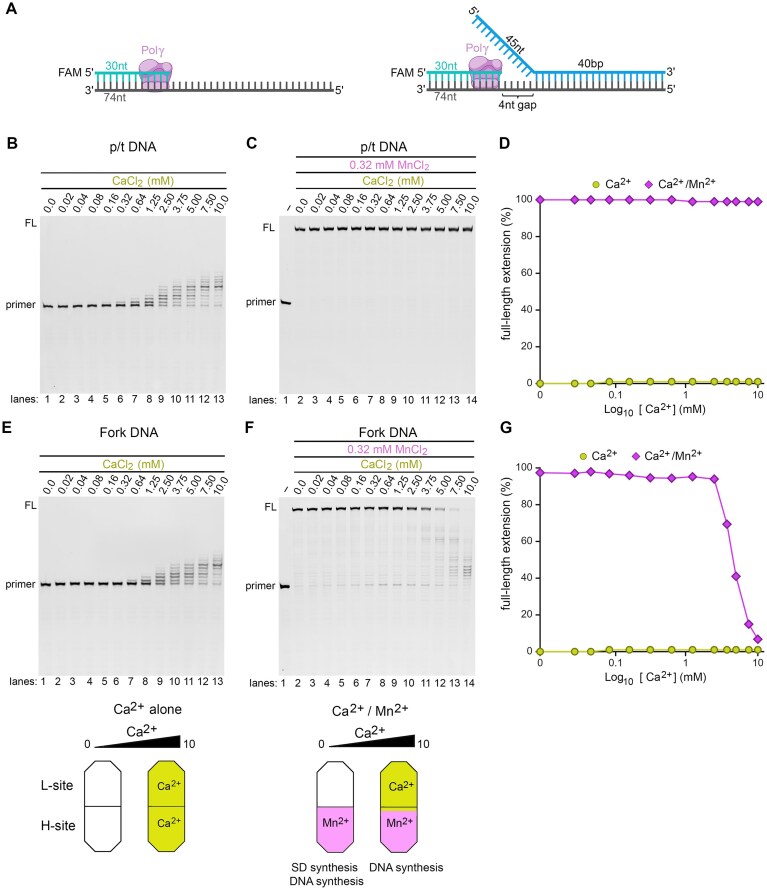
Pol γ strand displacement synthesis in the presence of mixed divalent metal ions. (**A**) p/t and fork DNA substrates used in this assay. Primer extension on a p/t DNA substrate in the presence of 0 to 10 mM Ca^2+^ without (**B**) and with a constant 0.32 mM Mn^2+^ (**C**); (**D**) quantification of (B, C) panels; Pol γ SD activity on the fork DNA in the presence of 0 to 10 mM Ca^2+^ without (**E**) and with 0.32 mM Mn^2+^ (**F**); and (**G**) quantification of (E, F) panels.

We first assayed Pol γ activity in the presence of Ca^2+^ (0.02–10 mM) alone and found the polymerase showed low activity on p/t or fork DNA (Fig. 4A, B, and E). We then added 0.32 mM Mn^2+^ that enables strand displacement to the assay. Our working model is that if the Pol activity diminishes, Ca^2+^ has displaced Mn^2+^ in the H-site; if Pol activity is unchanged and strand displacement diminishes, then Ca^2+^ has occupied the L-sites. To eliminate potential contamination by other metal ions, 1 mM EDTA was added to a 100 mM CaCl₂ stock solution of 99.98% purity.

On the p/t DNA, in the presence of Ca^2+^/Mn^2+^, Pol γ extended the primer to P + 44, nearly identical to with Mn^2+^ alone and different from Ca^2+^ alone (compare Fig. 4B–D with Fig. 1D), suggesting that even at maximum concentration (Ca^2+^/Mn^2+^ molar ratio = 30), Ca^2+^ cannot replace Mn^2+^ in the Pol site. However, on the fork DNA, Pol γ strand displacement synthesis was observed at Ca^2+^/Mn^2+^ molar ratios up to 12 and decreased when the ratio exceeded 15 (Fig. 4F and G). The reduced activity is likely due to Ca^2+^ occupying the L-sites, which can also inhibit DNA unwinding.

In the mixture of Mg^2+^ (0.64 mM) and Ca^2+^ (0–10 mM), the Pol activity began to decrease at substoichiometric Ca^2+^/Mg^2+^ molar ratio of 0.5, suggesting that Ca^2+^ has occupied the H-sites with a higher affinity than Mg^2+^, and the strand displacement activity diminished at Ca^2+^/Mg^2+^ molar ratio as low as 0.25 (Supplementary Fig. S6). These results imply that only Mg^2+^ or Mn^2+^ in the H-site can support strand displacement, whereas L-sites occupied by Mg^2+^, Mn^2+^ or Ca^2+^ all inhibit DNA unwinding. The experiments suggested affinities of metal ions to the H-sites follow the order of Mn^2+^>Ca^2+^>Mg^2+^, and Mn^2+^>Mg^2+^>Ca^2+^ to the L-sites.

### Localization of metal ion binding sites

Pol γ Mg^2+^, Mn^2+^, and Ca^2+^ binding sites were predicted using program MIB2 [59]. Scores were computed for each amino acid as confidence of its metal binding. Using criteria of scores greater than 1.0 that resulted in prediction accuracy for Mg^2+^, Mn^2+^, and Ca^2+^ of 94.6%, 95.0%, and 94.1%.

One hundred forty-four amino acids in Pol γA are involved in Mg^2+^ binding, 125 in Mn^2+^ binding, and 138 in Ca^2+^ binding. The highest scores are catalytic residues D^890^ and D^1135^ in the Pol site, D^198^ and E^200^ in the exo site. The predictions are consistent with structurally identified metal ion sites and our biochemical assays, lending high confidence to the prediction (Supplementary Table S2).

**Figure 5.**
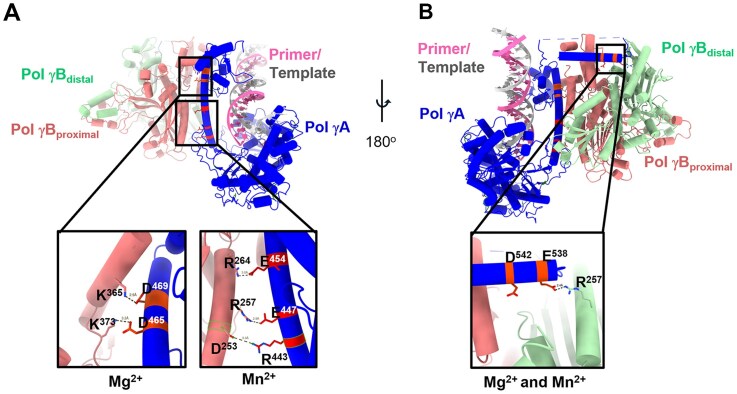
Computationally predicted Mg^2+^ and Mn^2+^ binding sites at subunit interface. Pol γ holoenzyme comprising the Pol γA catalytic subunit (blue) and dimeric Pol γB accessory subunit with proximal (pink) and distal (green) monomers, (**A**) the zoomed-in view of metal-binding sites between the Pol γA thumb and Pol γB proximal monomer C-terminal domain (CTD), and (**B**) metal binding sites between Pol γA AID domain and Pol γB distal monomer CTD.

As catalytic subunit Pol γA alone cannot carry out strand displacement synthesis (Supplementary Fig. S2), and Pol γA–Pol γB association is essential for strand displacement synthesis, we thus focused on the highly scored Mg^2+^ and Mn^2+^ bound at the Pol γA–Pol γB subunit electrostatic interface, as they could alter the stability of the holoenzyme. Three areas are identified: (i) the Pol γA AID subdomain that harbors two predicted binding sites for both Mg^2+^ and Mn^2+^, Glu^538^, and Asp^542^ (Fig. 5B). Glu^538^ forms electrostatic interaction with Pol γB distal monomer R^257^; (ii) the Pol γA thumb subdomain that contains Mg^2+^ potential binding sites at D^465^ and D^469^ that interact with Pol γB proximal monomer at K^373^ and K^365^, respectively (Fig. 5A); and (iii) four Mn^2+^ sites at R^443^, E^447^, and E^454^ that interact with Pol γB proximal monomer R^264^, R^257^, and D^253^, respectively (Fig. 5A). Binding to positively charged metal ions would weaken the electrostatic interactions at subunit interface (Supplementary Fig. S7), which in turn could reduce holoenzyme strand displacement.

We carried out dynamic cross-correlation analysis to probe metal ion-induced enzyme dynamics. The difference in cross-correlation between 20 mM and 1 mM Mg^2+^ showed that metal concentration induced distinct dynamic coupling and interdomain coordination. At 1 mM Mg^2+^, the finger domain showed increased anti-correlated motion with the thumb, palm, or exo domains (Supplementary Fig. S8B, purple circles), whereas the palm and thumb showed increased correlation with the exo than the 20 mM Mg^2+^ system (Supplementary Fig. S8A, green circles).

### Processivity of Pol γ strand displacement activity

**Figure 6.**
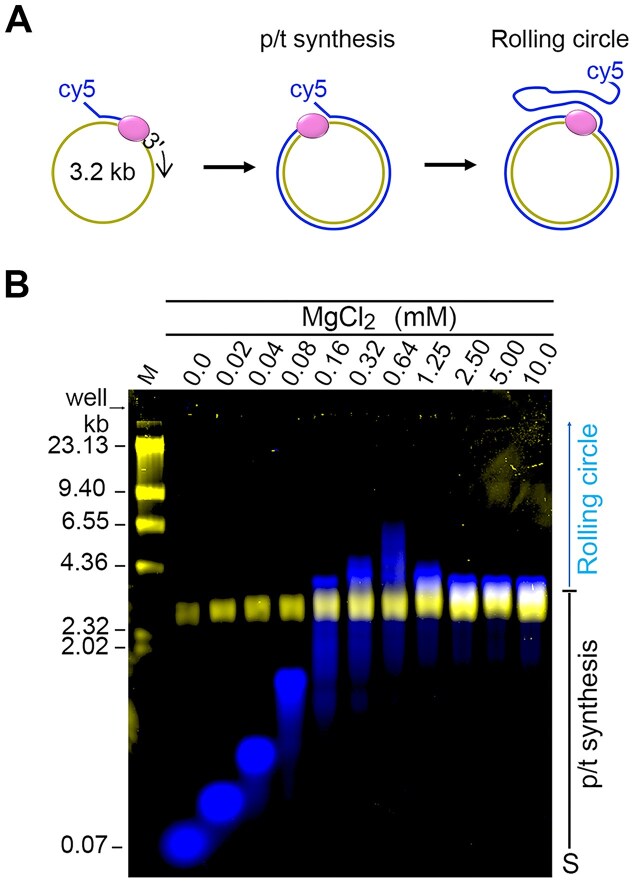
Metal-dependent rolling midi circle synthesis. (**A**) Scheme of the midi circle substrate constituted of a 5′-Cy5 71-nt primer and a circular ssDNA template, (**B**) products from Pol γ DNA rolling circle synthesis resolved on an alkaline gel (0.8%), scanned using dual-wavelength mode. The circular single-stranded DNA template was visualized with a 488 nm scan for SYBR stain (yellow), and the 5′-Cy5-primer and its extension products are visualized with a 635 nm scan (blue).

Pol γ *midi-rolling circle* synthesis was carried out using a 3200-nt single-stranded circular DNA annealed to a 5′-Cy5-71-nt primer (Fig. 6A). The primer extension up to 3.2 kb was synthesized on the ss-template (p/t synthesis) and the length beyond is the product of strand displacement (rolling circle).

Because Pol γ DNA-binding site is ∼25 bp, the synthesis product of the 3.2 kbp and elongating ssDNA is a 300 000-fold excess to the binding site, thus serving as an effective competitive DNA for the dissociated Pol γ and disrupting its rebinding to the fork junction under our experimental conditions. The length of product is greater than 3.2 kb, therefore, represents processivity of Pol γ strand displacement synthesis. The assays were conducted in the presence of 0–10 mM Mg^2+^. Like on the short DNA, Pol γ synthesis on the 3.2 kb ss-template was observed at 0.02–0.08 mM Mg^2+^. Beginning at 0.16 mM Mg^2+^, strand displacement synthesis was observed, and the maximum product length reached 6.5 kb at 0.64 mM (Fig. 6B) and decreased at higher [Mg^2+^]. This indicates that the strand displacement synthesis processivity of Pol γ is 3.3 kb, comparable to that on the single-stranded template [44].

### Cryo-EM structure of Pol γ complexed to a fork DNA revealing elements/origin of strand displacement

**Figure 7.**
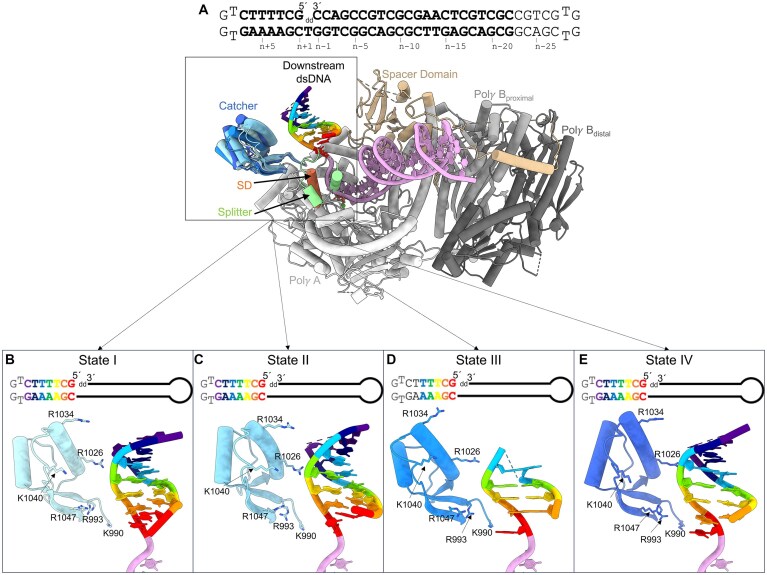
Cryo-EM structures of Pol γ strand displacement complex. (**A**) Dumbbell DNA substrate used in cryo-EM studies. Pol γ-dumbbell DNA complexes superimposed by aligning the Pol site, showing the different conformations of the Catcher domain (different shades of blue). The Catcher, the Splitter, and strand displacement (SD) domains are located near the downstream DNA (rainbow color). (**B**–**E**) Close-up views of conformations of the Catcher domain and DNA in different states. (B) dsDNA in full-duplex form (State 1), (C) the n + 1 base pair (bp) buckled (State II), (D) the n + 1 bp unwound (State III), and (E) the n + 1 bp unwound and the n + 2 bp buckled (State IV).

To elucidate the structural mechanism by which Pol γ unwinds DNA, we determined a cryo-EM structure of the Pol γ ternary complex bound to a 77-nt DNA substrate. The DNA was folded into a dumbbell with a 27-bp upstream and a 7-bp downstream hairpin, separated by a single-nucleotide gap (dT). The primer strand was terminated with 3′-dideoxycytosine (ddC), and the correct incoming nucleotide, dATP, was included (Fig. 7A). Although the enzyme and DNA were of high homogeneity in solution, four conformers were dissected to 2.62–3.23 Å resolutions (Fig. 7B-E and Supplementary Fig. S1). Structural data collection and refinement statistics are shown in Supplementary Table S3.

**Figure 8.**
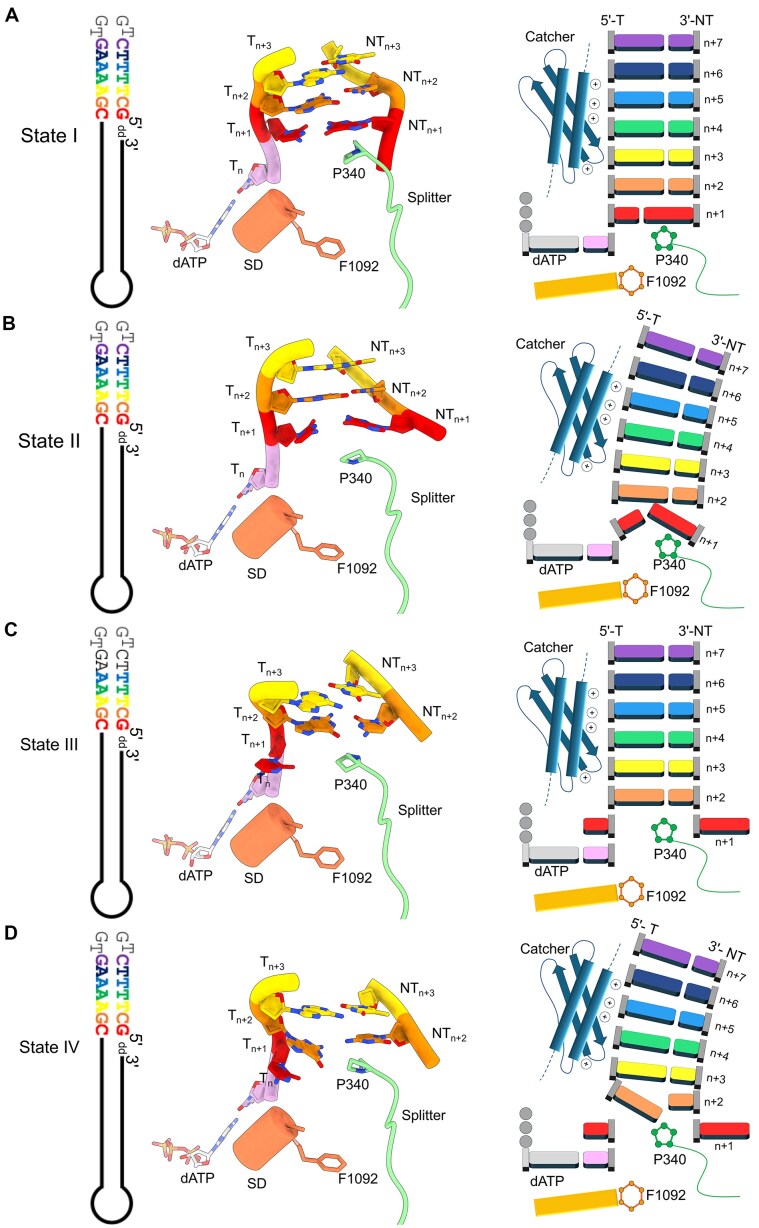
Unwinding mechanism of the downstream dsDNA in Pol γ. (**A**–**D**) Dumbbell DNA substrate with visible downstream duplex shown in corresponding color as the cryo-EM structure (*left*). Pol γ adopts a pre-translocation conformation, where a dATP forms base pair with the template T_n_ in the Pol site (*right*). As no catalysis was permitted, no DNA translocation occurred. Although near the downstream, the SD helix and Splitter loop do not change conformation with the change of dsDNA unwinding state, whereas the Catcher domain undergoes compensatory changes with the dsDNA. In states II and IV, n + 1 and n + 2 base pairs are buckled against Pro^340^. (*right*) cartoon representation of downstream duplex unwinding along with the correlated movement of the catcher and the downstream duplex.

All Pol γ-DNA conformers assumed the replication mode, where the 3′-end of the dumbbell and dATP were in the Pol site (Supplementary Fig. S9). While the upstream dsDNA and Pol γ were nearly superimposable, the Catcher domain (Residues 994–1049) and downstream dsDNA exhibited structural diversity (Fig. 7B–E). The downstream 7-bp dsDNA was captured in various unwinding states: a fully base-paired duplex (State I), the buckled *n + 1* base pair (State II), the unwound n + 1 bp (State III), and the unwound n + 1 and buckled *n + 2* base pair (State IV) (Fig. 8 A–D). Despite different degrees of unwinding, the positions of the template T_n+1_ and T_n+2_ backbone are invariant, adopting identical positions as those in the ss-template complex [21, 60]. The backbone of T_n+1_ and T_n+2_ rests on a loop (G^956^A^957^G^958^) connecting O- and O1-helices (Supplementary Fig. S9), stabilizing the dsDNA without specific interaction. The downstream dsDNA in State I–IV exhibited a rolling motion around T_n+1_ and T_n+2_ of ~20°. As the primer contains a 3′-ddC, no catalysis would occur; the structural ensemble, therefore, represents the dynamics of the downstream duplex at the ground state. The structures showed that the n + 1 and n + 2 base pairs of downstream dsDNA are less stable and can unwind without any external force. Our cryo-EM results are consistent with observations from NMR studies where 2–3 bp could be spontaneously unwound [15].

Three protein elements near the downstream dsDNA fork could potentially participate in DNA unwinding (Fig. 8): The helix-turn-helix Catcher domain (residues 994–1049) (Fig. 7B–E), which is termed following the nomenclature of yeast mitochondrial DNA polymerase, Mip1 [61]; the Splitter helix and the connecting loop (residues 301–345) that could be involved in proofreading [60]; and a strand displacement helix (residues 1087–1100) in the fingers domain (Figs 7A and B and 8).

The Catcher domain displayed a polarized charge distribution, the helix facing the downstream DNA is positively charged; with residues K^1027^, R^1026^, R^1030^, R^1034^, K^1035^, and R^1040^ interacting primarily with the template strand of the downstream dsDNA (Fig. 7C–E), and the helix on the opposing side is negatively charged, containing D^999^, E^1000^, E^1002^, and E^1007^ (Supplementary Fig. S10C). The Catcher domain is less ordered in the Pol γ complexed with a single-stranded template and only emerged in the structure containing dsDNA template. In the dumbbell complexes, the Catcher undergoes compensatory conformational changes with the downstream DNA (Fig. 8 and Supplementary Fig. S10A and B), suggesting their mutual stabilization. Specific electrostatic interactions were seen between R^1049^ and template nucleotides T_n+2_ and T_n+3_, R^1029^ with template T_n+2_ and non-template NT_n+3_ (Fig. 7B–E). The structure of the Catcher domain is homologous with other DNA/RNA-binding proteins, such as *S. cerevisiae* translation machinery-associated protein 64 (RMSD = 2.13 Å), *B. subtilis* RacA DNA-binding protein (RMSD = 2.41 Å), and *H. sapiens* mdm2 protein (RMSD = 2.49 Å).

The Splitter is conserved among mitochondrial DNAPs, but the length of connecting loop in human Pol γ is longer than others. Deletion of the W347–L356 in conjunction with A467T was found in patients with Alpers syndrome. Out of 24 children who had the deletion, 21 had Alpers syndrome, comprising hepatoencephalopathy and mtDNA depletion [62].

The tip of the fingers helix contains a bulky phenylalanine, F^1092^ (Fig. 8). While the helix is slightly off the axis of the downstream DNA, its structural homology to Mip1 and T7 RNAP, together with the flexible downstream duplex location, suggests the helix could still be involved in dsDNA strand separation. The corresponding residue F^848^ in Mip1 was confirmed for the strand displacement activity by mutational studies [61].

Although the above human Pol γ protein elements are potentially involved in strand displacement, we believe that they likely facilitate strand separation but are not the primary energy source. In Pol γ, the connecting loop of the Splitter helix is located near the n + 1 base pair of the downstream duplex. Though partially disordered, the loop’s location implies it could be involved in strand separation. Motifs Pro^340^–Ala^341^ and Asp^756^–Asn^761^ restricted the entrance to the Pol site to the dimension suitable only for a single-stranded template, thus facilitating strand unwinding while the energy source arises from dNTP binding and incorporation. The presence of the elements might be necessary but insufficient for Pol γ strand displacement synthesis, as the activity is more intricately regulated in the human enzyme (see the “Discussion” section below).

## Discussion

Due to the division of labor within a DNA replisome, in which helicases unwind duplex DNA and DNA polymerases synthesize new strands, replicative DNA polymerases typically exhibit limited strand-displacement activity on double-stranded DNA templates. However, this division of labor in the human mitochondrial replisome remains unclear. Twinkle helicase alone exhibits minimal unwinding activity, whereas the Twinkle–Pol γ complex displays robust strand-displacement synthesis, leading to the hypothesis that Pol γ contributes significant motor function within the replisome.

Pol γ strand displacement synthesis is particularly important for human mtDNA asymmetric replication, in which the H- and L-strands are replicated in an uncoupled and asynchronous manner, in contrast to canonical strand-coupled synthesis. In this model, H-strand synthesis begins from a primer generated by mitochondrial RNA polymerase at the origin OriH and proceeds to replicate toward the other origin, OriL, generating an ∼11 kb single-stranded DNA (D-loop) for replication of the L-strand. The Pol γ strand displacement activity may be critical in three steps: (a) transition from RNA transcription to DNA replication at OriH. The transcription bubble (8–10 nt) is too small for Twinkle binding, and Pol γ strand displacement synthesis can expand the bubble to a sufficient length for Twinkle loading; (b) synthesis on the 11 kb ssDNA, where Pol γ strand displacement synthesis can resolve numerous secondary structures, including hairpins and G-quadruplexes, on the D-loop; and (c) upon completion of DNA synthesis, Pol γ strand displacement synthesis can displace the RNA primer before ligation of the two DNA ends.

Previous studies reported inconsistent strand-displacement activity for Pol γ, ranging from only 1–2 nt to ∼37 nt [9, 18]. The limited activity has been attributed to inefficient prevention of strand reannealing following displacement of the non-template strand [18]. We noted substantial differences in experimental conditions among these studies, particularly in divalent metal ion concentrations, and therefore investigated the effects of metal ions on Pol γ activity. We examined the effects of Mg²⁺ and Mn²⁺ on Pol γ activity and found that Pol γ exhibits a clear metal ion concentration-dependent strand displacement activity. Under physiological concentrations of Mg²⁺ and Mn²⁺, Pol γ can carry out strand displacement synthesis with high processivity (∼3–4 kb).

### Dual roles of metal ions in Pol γ DNA synthesis and unwinding

We hypothesize that Pol γ contains at least two classes of metal-binding sites: high-affinity (H) sites that support DNA synthesis and strand displacement, and low-affinity (L) sites that suppress DNA unwinding without affecting DNA synthesis. Our results indicate that only catalytically competent metals (Mg²⁺ and Mn²⁺) bound at the H-sites support strand-displacement synthesis. In contrast, Mg²⁺, Mn²⁺, or catalytically inactive Ca²⁺ bound at the L-sites inhibit DNA unwinding.

While the affinity of Mg²⁺ bound to protein can be determined, such as Mg^2+^ binding to *E. coli* DNA polymerase I (Pol I) using electron paramagnetic resonance (EPR), which identified four high-affinity and ∼20 low-affinity sites [63], the precise locations of the protein-bound metal ions can only be unequivocally determined by multiwavelength anomalous dispersion method [64]; unfortunately, the energy edge for Mg^2+^ anomalous signal collection cannot be reached currently. Therefore, the locations of the different affinity Mg^2+^ and Mn^2+^ sites remain hypothetical until further development of technology. It is safe to assume catalytic metal ion sites for Pol and Exo are among the high-affinity sites.

Mitochondria lack a dedicated primase; instead, RNA primers are synthesized by the mitochondrial RNA polymerase, POLRMT [65, 66]. These primers can be hundreds of nucleotides long, and their removal is essential for mtDNA stability [67, 68]. Mechanism of RNA primer removal in mitochondria has not been completely elucidated. RnaseH1, together with EXOG and FEN1, are all shown to participate in this process [69–72]. We showed here that Pol γ can efficiently displace RNA from RNA/DNA hybrids, suggesting its potential role in RNA primer removal and analogous to *E. coli* DNA Pol I [73].

### Exonuclease activity and strand-displacement synthesis

A contributing factor to the limited strand-displacement activity of Pol γ is its 3′–5′ exonuclease activity, a phenomenon also observed in other DNA polymerases, including human Pol δ, Pol ε, and bacteriophage T7 DNA polymerase [16, 17, 51]. In these enzymes, strand-displacement activity reflects a balance between polymerase and exonuclease functions.

We therefore examined whether metal-dependent stimulation of strand displacement arises from suppression of exonuclease activity. Although exonuclease activity decreases with increasing metal ion concentration, Pol γ retains ∼60% (Mg²⁺) and ∼90% (Mn²⁺) of exonuclease activity under conditions that promote strand displacement, indicating that proofreading remains largely intact.

Moreover, the Pol γ^exo–^ variant remains sensitive to metal ion concentrations. Notably, Pol γ^exo–^ exhibits identical Mn²⁺ dependence as wild-type Pol γ, demonstrating that metal-stimulated strand displacement is not solely due to suppression of exonuclease activity. Although all catalytic activities of Pol γ reside in the catalytic subunit Pol γA, the subunit alone does not support strand-displacement synthesis under any tested metal ion conditions. Only the holoenzyme, formed by association of Pol γA with the accessory subunit Pol γB (which lacks intrinsic catalytic activity), exhibits robust strand displacement. This suggests that metal binding to Pol γA alone is insufficient and that additional metal-binding sites likely exist at the Pol γA–Pol γB interface or within Pol γB.

Computational analysis identified high-confidence metal-binding sites at two Pol γA–Pol γB interfaces, potentially disrupting electrostatic interactions and weakening subunit coupling (Fig. 5 and Supplementary Fig. S7). Molecular dynamics simulations further show increased structural rigidity at high Mg²⁺ concentrations (20 mM) compared to physiological levels (1 mM), with reduced root-mean-square fluctuations in the palm, thumb, and finger domains. These findings suggest that metal binding at L-sites increases enzyme rigidity, which is unfavorable for strand displacement but does not impair polymerase activity.

### Structural and molecular model of strand-displacement synthesis

DNA polymerases capable of strand-displacement synthesis are found in both Pol A and Pol B families [74, 75]. Structural analysis reveals diversity in the architecture of the downstream DNA entry site.

**Figure 9.**
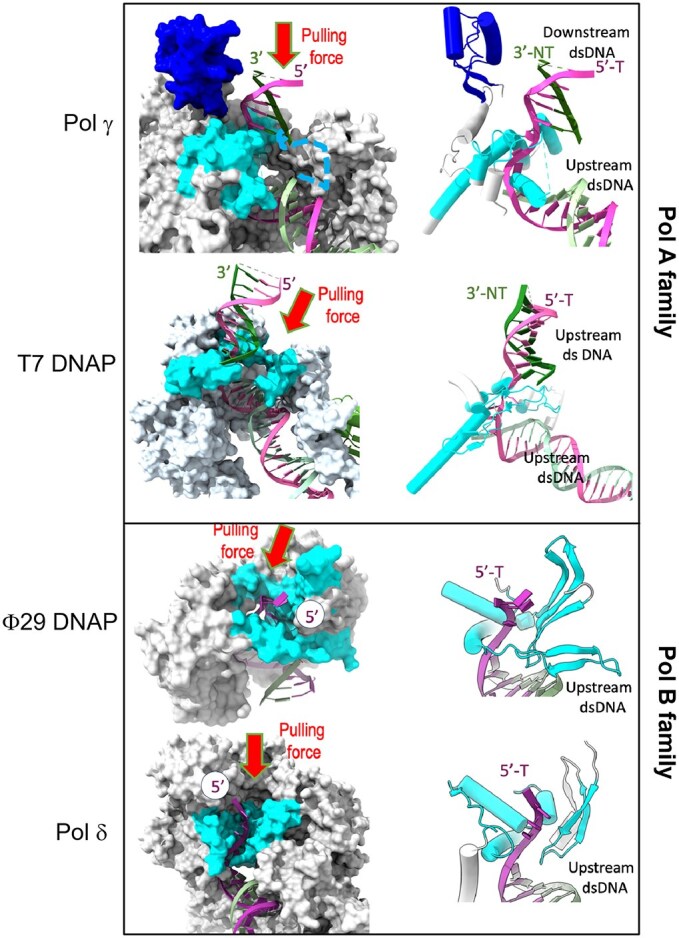
Diverse structural features of DNAPs with strand displacement capability at the Pol site entrance. Pol A family members, Pol γ and T7 DNAP, showed a “C-shaped” Pol site entrance that facilitates displacement of the non-template (NT) strand, while the corresponding protein elements are not conserved. Pol B family members, phi29 DNAP (PDB: 2PYJ), and Pol δ (PDB: 7KC0). The Pol site entrance in phi29 DNAP is completely enclosed, forming an “O-shape,” while in Pol δ is “C-shape,” and the structural components of the entrance are not strictly conserved. The dashed line in Pol γ represents the disordered portion of the Splitter domain.

Among Pol A family members—including Pol γ, Mip1, Pol I, and T7 DNA polymerase—a common feature is a “C-shaped” polymerase-site entrance that accommodates the template strand while displacing the non-template strand (Fig. 9, top). Similarly, Pol B family members—including Pol δ, Pol ε, and RB69 [76–78]—also exhibit C-shaped architectures. In contrast, phi29 DNA polymerase, which displays exceptionally strong strand-displacement activity, forms a closed “O-shaped” entrance that fully encircles the template strand and excludes the non-template strand (Fig. 9, bottom).

Despite these functional similarities, the structural elements forming the polymerase entrance are not conserved. In the Pol A family, T7 DNA polymerase contains a positively charged β-turn–β hairpin (residues 575–590) that contributes to strand displacement. Mutations of key basic residues (K587, K589, R590, R591) significantly impair DNA synthesis and reduce viral growth (∼10-fold) [80]. The corresponding region is occupied by a helix in [79] Pol I, part of the Splitter domain in Pol γ (Fig. 9), and is absent in Mip1 [61].

In the Pol B family, the polymerase entrance consists of β-sheets and α-helices with no structural homology to Pol A enzymes. In RB69 DNA polymerase and Pol δ, only a single β-sheet pair is present, whereas phi29 DNA polymerase contains a triple β-sheet structure and an additional pair that fully encloses the active site.

Based on these observations, we propose a model in which the energy driving strand displacement originates from nucleotide incorporation. Each dNTP addition provides >10 kcal/mol of free energy from binding and phosphodiester bond formation [81], exceeding the energy required to unwind a base pair (∼2–3 kcal/mol). Following incorporation, polymerase translocation generates a pulling force on the template strand. Because the polymerase entrance sterically restricts passage to single-stranded DNA, this force promotes displacement of the non-template strand (Fig. 9).

## Conclusion

Our study provides a structural and mechanistic framework for understanding strand-displacement synthesis by human Pol γ and its regulation by divalent metal ions. Mg²⁺ and Mn²⁺ play dual roles in catalysis and regulation of DNA unwinding. Under physiological metal ion concentrations, Pol γ exhibits robust, highly processive strand-displacement activity, reconciling previously inconsistent observations.

## Supplementary Material

gkag720_Supplemental_File

## Data Availability

Cryo-EM density maps and atomic coordinates have been deposited using the Worldwide Protein Data Bank (wwPDB) OneDep System. Pol γ strand-displacement ternary complexes are available under the following accession codes: State I (EMD-72478) (PDB: https://doi.org/10.2210/pdb9y4c/pdb), State II (EMD-72479) (PDB: https://doi.org/10.2210/pdb9y4d/pdb), State III (EMD-72480) (PDB: https://doi.org/10.2210/pdb9y4e/pdb), and State IV (EMD-72481) (PDB: https://doi.org/10.2210/pdb9y4f/pdb).
